# Methodology for linking Ryan White HIV/AIDS Program Services Report (RSR) client level data over multiple years

**DOI:** 10.1371/journal.pone.0237635

**Published:** 2020-08-21

**Authors:** Julia Zhu, Miranda Fanning, Laura Sheehan, Kerry Grace Morrissey, Stan Legum, Sigurd Hermansen

**Affiliations:** 1 Health Resources and Services Administration, HIV/AIDS Bureau, Division of Policy and Data, Rockville, Maryland, United States of America; 2 Accenture Federal Services LLC, Arlington, Virginia, United States of America; 3 Westat Inc., Rockville, Maryland, United States of America; Washington University in Saint Louis, UNITED STATES

## Abstract

**Background:**

The Health Resources and Services Administration’s (HRSA), HIV/AIDS Bureau (HAB) is responsible for leading the nation’s efforts to provide health care, medications, and support services to low-income people living with HIV through the Ryan White HIV/AIDS Program (RWHAP). The RWHAP funds and coordinates with cities, states, and local community-based organizations to deliver efficient and effective HIV care, treatment, and support services for over half a million vulnerable people living with HIV (PLWH) and their families in the United States. The annual RWHAP Services Report (RSR) is an important source of information for monitoring RWHAP’s progress towards National HIV/AIDS Strategy goals. Since 2010, HRSA HAB has used the annual client-level RSR data to monitor program-related outcomes, conduct program evaluations, understand service provision, and conduct extensive analysis on disparities in viral suppression and retention in HIV care. HRSA HAB receives annual RSR submissions from RWHAP recipients and sub-recipients. However, the de-identified nature of the data limits HRSA HAB’s ability to expand beyond year-to-year analyses and conduct additional analyses to evaluate outcomes for clients who are seen in multiple years. The current paper describes the development and validation of a method to link RSR client-level records across multiple data years.

**Methods and findings:**

Using seven RSR reporting years of data (2010 to 2016), we applied a Fellegi-Sunter (F-S) linkage model that used client demographic characteristics and their providers’ geographic locations to calculate matching weights for each record pair based on estimated agreement and disagreement conditional probabilities across RSR years. To validate our methodology, we conducted an internal sample review and external validation to assess the level of accuracy of the linkage, and the extent to which the linked data set corresponds accurately to clinical records of individual clients. The linkage result yielded 70 to 80 percent year-to-year client carry-over rate over seven years of the RSR data; 96 percent linkage ratio from the internal sample review and 79.9 to 94.2 percent of provider network client carry- over rate per year from the external validation.

**Conclusions:**

This methodology addresses a gap in data analysis capabilities by allowing HRSA HAB to link RWHAP clients across reporting years. Despite weak identifying information and lack of continuity of service reporting, the longitudinal linkage improves HRSA HAB’s ability to evaluate the patterns of viral suppression and monitor service utilization over time for individuals who receive services in multiple years. These analyses will support future analytic activities in understanding the impact and outcomes of the RWHAP, and will assist HRSA HAB in monitoring progress toward meeting National HIV/AIDS Strategy goals. For those looking for ways to assess health services data, the F-S unsupervised method combining weak identifying attributes and geographic proximity offers practical solutions to the problem of linking de-identified information about individuals across multiple years and improving longitudinal research.

## Introduction

The Health Resources and Services Administration’s (HRSA), HIV/AIDS Bureau (HAB) is responsible for leading the nation’s efforts to provide health care, medications, and support services to low-income people living with HIV through the Ryan White HIV/AIDS Program (RWHAP) [1]. The RWHAP funds and coordinates with cities, states, and local community-based organizations to deliver efficient and effective HIV care, treatment, and support services for over half a million vulnerable people living with HIV (PLWH) and their families in the United States [2, 3]. As a condition of this funding, RWHAP grant recipients and providers are required to report annual data on clients served, services provided, and expenditures to HRSA HAB. HRSA HAB uses these data to monitor program-related health outcomes, evaluate program activities, track service utilization, and assess disparities in viral suppression and retention in HIV care.

RWHAP-funded recipients and service providers submit annual data in the RWHAP Services Report (RSR) -, a client-level data system that captures information on the characteristics of RWHAP grant recipients, service providers, and clients. Because HRSA HAB is statutorily prohibited from the collection of Personal Identifiable Information (PII), such as name, date of birth and social security number, client-level data are de-identified by RWHAP recipients and/or service providers prior to submission [4]. Clients within the same service provider or provider’s network are uniquely identified using a forty- or forty-one character encrypted Unique Client Identifier (eUCI40 or eUCI41).

The eUCI40 is an encrypted client identification code generated from the following elements: the first and third characters of a client’s first and last names, full date of birth, and gender code. This encrypted ID is a string with length 40; thus, it is called the eUCI40. If two or more clients have identical eUCI40s, service providers will add a character at the end of the eUCI40s to create 41-digit eUCIs (eUCI41) to distinguish between clients (see < https://targethiv.org/sites/default/files/file-upload/resources/eUCI_Application_User_Guide_Dec_2014.pdf>). The choice of this additional character is not universal across providers; each eUCI40 or eUCI41 is only unique for clients within the same service provider and year. Unfortunately, this process creates analytic challenges in the RSR data for HRSA HAB because it introduces the potential for obtaining duplicate records across providers and years as well as limits the ability to link client data across time.

HAB previously developed a single-year de-identification duplication methodology to uniquely identify clients within a single year across providers and provider networks. The approach uses probabilistic matching to assess the likelihood that two clients are the same, given equal values on common data elements (i.e., race, ethnicity, and housing status). Records determined to belong to the same client are provided with a 2-digit suffix, such as “00”, “01”, “02”, that replaces the provider assigned character at the end of the eUCI40 (i.e., eUCI42). De-duplication of RSR data is performed independently each year by HRSA HAB. As a result, the eUCI42 cannot be used to identify the same client across years. This inability to link data across years limits HRSA HAB’s ability to assess longitudinal trends in HIV viral suppression and retention in care as well as understand the factors associated with these outcomes.

To address this limitation, HRSA HAB sought to develop and validate a method to link RSR client-level records across multiple data years. In this paper, we describe the development of a longitudinal record linkage methodology for RSR data and provide evidence for the validity of this approach. We also discuss the implications for using this methodology in other settings.

## Methods

### Ethics statement

The current analyses involved the analysis of existing de-identified data for which investigators cannot link back to participants and, as such, is exempt from human subjects review (U.S. Department of Health and Human Services (HHS) regulation 45 CFR 46.101(b)).

### Selection of a longitudinal data linkage algorithm

To identify the most appropriate data linkage methodology related to the health care field and the study of people living with HIV (PLWH), we conducted a literature review of over 400 peer-reviewed articles, reports and publications from four major scientific research databases on the web (PubMed, PubMed Central, Google Scholar, and Lex Jansen). The inclusion criteria limited searches to: 1) publications since 2000, except for key or highly relevant publications published prior to 2000; 2) English language publications; 3) record linkage/matching methods and strategies, data integration, object identification in statistics, computer science, medicine, database management, and web technology search terms; and, 4) natural key linkage of disparate data sources and methodologies aimed at identifying and de-duplicating data drawn from the PLWH and other special populations.

The literature on data linkage methods (i.e., “data integration” and “entity resolution”) includes many examples of the standard F-S probabilistic linkage model [5], its extensions (e.g., Expectation-Maximization), latent class, and statistical/machine learning (support vector machines, random forests, and deep learning) classifiers. For the HRSA HAB longitudinal linkage, only weak (in fact, mostly de-identified) attributes of persons were available for training a classifier.

We divided the prospective data linkage methods into two groups: supervised, requiring a substantial “truth set” of known client record links and non-links; and unsupervised, without that requirement. Based on the literature review, we selected the unsupervised F-S model because the methodology generally provides robust and reliable linkage results in the absence of a truth set, and it is the methodology commonly cited in the literature reviewed [5–47]. In addition, the use of the F-S unsupervised method has proven successful in contexts similar to that of HRSA HAB longitudinal linkage [6].

### Model overview

The F-S model is a probabilistic approach to solving record linkage problems based on conditional probabilities. Although it comes from an earlier era, it has much in common with more recent classical and Bayesian statistical and machine learning classifiers in that it employs an ensemble of a weak classifiers and prior distributions of true and false signals from classifiers. This method classifies all record pairs that come from two (or more) data sources into three independent and mutually exclusive groups: true matches, non-matches, and uncertain matches. Record pairs are classified based on a conditional probability model that calculates both the likelihood ratio and the match score that the record pairs represent the same individual based on the summarized and weighted level of agreement or disagreement between values of selected variables. Weights represent the estimated frequency of a match of a variable’s values given a true match divided by the frequency of a true match given a non-match of the variable’s values. In the absence of more precise conditional probabilities, a model with a weight of 0.9/0.1 for all variables will work well enough.

Under the F-S Model, the classification decision involves setting two threshold weights: an upper threshold above which a record pair is classified as a match, and a lower threshold below which a record pair is classified as a non-match. The choice of these selection thresholds aims to minimize both the linkage errors and the number of pairs with an indeterminate status between the two thresholds [5]. In practice, many applications use a single threshold for classification to achieve the desired error levels [7–8]. Our linkage methodology adopted the single threshold for classification. Pairs at or above the classification threshold were declared as a match; those below the classification threshold were declared as a non-match. In a longitudinal setting, we began with the eUCI40 linkage results for a base year (2010) and extended those to the next year, and then to each subsequent year. This progression linked records with similar attributes and proximity, and it de-linked records with differences in similarity and proximity.

### Matching variable selection

The matching variables consisted of a set of personal attributes and geographic codes of service providers’ addresses that were used to link client records in record comparisons in the F-S model. The selection criteria of matching variables from the RSR data included consistency and availability of each variable. An exploratory review of personal attributes that might serve to link client records preceded the final selection of variables for use in record comparisons. Within eUCI40 groups, client attributes carried over from one visit to a provider to the next visit, within and across years, and appeared consistent with eUCI40s assigned by that provider. Within the same geographic area, we also found that personal attributes within an eUCI40 group linked clients across different providers. Further, sequences of visits to providers by clients with very similar attributes showed potential for linking records belonging to a single client. In some instances, sequences of visits by clients ceased in one geographic area and appeared to resume in another area. The sequences of the eUCI40 IDs by provider showed continuous records in many cases, and gaps and breaks in others. Increases in continuity of the eUCI40 across years indicated correct linkage. Contemporaneous sequences of eUCI40 across years in different but proximate providers for a client provided even stronger evidence of correct linkage. These patterns suggest complementary roles of sets of identifying personal attributes and of geographical location in linking clients longitudinally.

A redeeming virtue compensating for weakly identifying personal attributes in RSR data turned out to be the relatively small number of RSR records in a typical eUCI40 group. Because different providers used the same rules to create the eUCI40 for a client, the eUCI40 groups carried over from provider to provider. Even though different clients could have the same eUCI40, the eUCI40 confined most clients to groups (or blocks) within which geographic and personal attributes could potentially distinguish one client from another.

The matching variables that were initially considered were race, ethnicity, housing status, poverty level, HIV status, HIV risk factors, provider, enrollment status, transgender status, geographic unit, first service year, HIV diagnosis year, death date, and first ambulatory care year. After examining each of the selected variables, we removed the variables that had a high rate of nonresponse, invalid values, or with a low information value for distinguishing true matches from non-matches. We also excluded variables that contributed to the eUCI40 generation (date of birth gender, and transgender status). In addition, we added a new variable Provider Core Based Statistical Area (PCBSA) [48] by geocoding the providers’ address and mapping the provider location to a metropolitan statistical area (MSA) [49]. Because RSR data not include client addresses, we used the PCBSA variable as a proxy for client location. The variables ultimately selected for RSR linkage were race, ethnicity, housing status, poverty status, HIV risk factor, HIV/AIDS status, provider ID, and provider service location: state and Core Base Statistical Area (CBSA) [49] codes.

Each matching variable carried a different weight in contributing evidence of distinguishing records. Some had more discriminative value than others. For example, agreement on provider, provider’s CBSA location code and state code contributed more evidence of a match than agreement on characteristics such as ethnicity or race.

### Matching algorithm

We first created pairs of client records from years 2010 (*A2010*) and 2011 (*B2011*) RSR data sets. The algorithm forms pairs of records in blocks (groups) sharing the same eUCI40s. The initial comparison was made on NA2010i * NB2011i number of pairs within each block in year 2010 data set (*A2010*) and 2011 data set (*B2011*), where *NA2010i* is the number of records in a block in data set *A2010*, *and NB2011i* is the number of records in the same block i in data set *B2011*.

Then the F-S model was applied to the linkage algorithm to calculate the matching weights for each variable based on the estimated agreement conditional probabilities (*m*) and the disagreement conditional probabilities (*u*). A starting estimate of *m* could be 0.9 for a name match in two records of the same person, and a *u* estimate of 0.05 for a name match between two different persons. The agreement weight was calculated as (log [*m*/*u*]) and the disagreement weight was calculated as (log [(1-*m*)/(1-*u*)]) for each matching variable. Agreement weights were generally positive and disagreement weights were generally negative.

For each record pair, the match score was calculated by adding the agreement weights for each matching variable that was in agreement and the disagreement weights for each matching variable that was in disagreement. Record pairs with agreement on multiple matching variables will have large positive match scores; record pairs with disagreement on most matching variables will have negative match scores. The matching score represents the likelihood of records belonging to the same individual, given the agreement or disagreement on the set of matching variables. The above approach for estimating the linkage parameters was an iterative refitting process. Each cycle merged an annual data set to the previous data set and formed a combined data set in the longitudinal series.

The formal F-S model specifies a comparison vector of variables v [1,J] and weights **w**[1,J;k] from data i ∈{1,2} having latent probabilities of k = {m_j_,u_j_) if there is a match or non-match of v_*j_, where match score s =

$$\sum_{v}(c_{ij})\times w_{jk=m}+(1-c_{ij})\times w_{ij=m}$$

$$where\;c_{ij\;}=1\;if\;(v_{1j}=v_{2j})\cup 0\;if\;(v_{1j}\neq v_{2j})$$

A threshold score determines the assignments of pairs of records to matched or unmatched classes. The selection of a threshold has a key role in data linkage of two data sets. All analyses were performed in SAS version 9.3 (SAS Institute Inc., Cary, North Carolina, USA).

### Threshold selection

**Fig 1:**
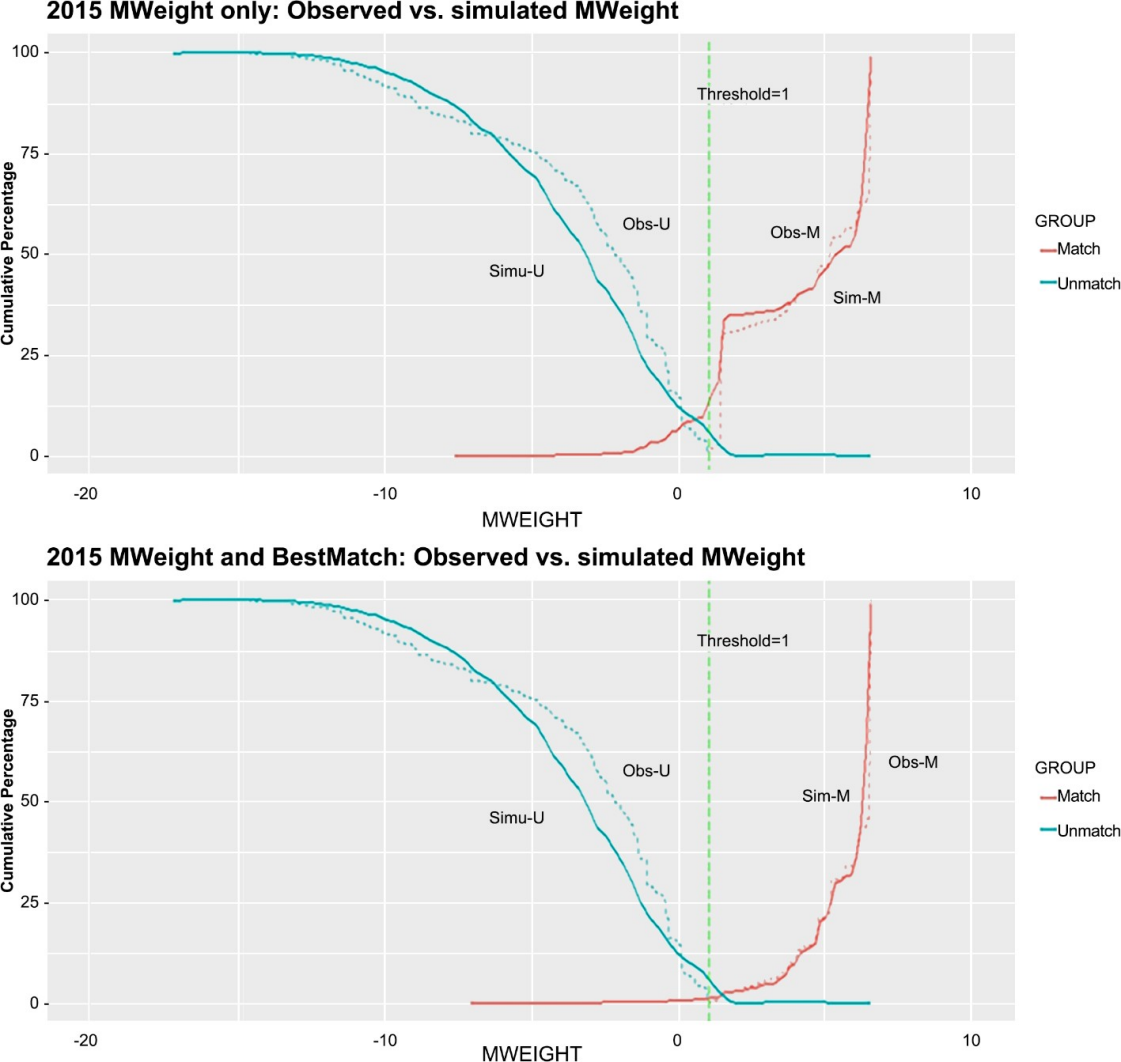
Cumulative distribution functions of the final match weight, 2015.

We selected the threshold for classification based on the distributions of match scores for true matches and non-matches from the pilot test on 2015 RSR data sets. We plotted both the observed frequencies and simulated frequencies to evaluate the estimated linkage error at the selection threshold and to minimize errors. Fig 1 shows the cumulative distribution functions (cdf) of the matched weights for the matched and unmatched pairs in Cycle 2015. The first plot shows the cdf for all matched pairs; the second plot shows the best pairs retained in 1:1 matching. Using the threshold at a match weight score of 1, the 2010–2015 pilot test yielded a 60.5 percent (695,985 record pairs) agreement on all matching variables of the matched pairs within eUCI40s. For individual match variables, there was 96 percent agreement for race, 99 percent agreement for the risk and provider MSA variables, and greater than 80 percent agreement for the other variables.

### Validation

We conducted sample review and external validation to identify potential errors, as well as to assess the level of accuracy of the linkage and the extent to which the linked data set corresponds to what was collected and captured within RWHAP clinics. In addition, we assessed evidence of improvements in the accuracy, coverage, and continuity in the longitudinal database.

#### Sample review

We reviewed a stratified client record sample from the RSR linked data set to assess the accuracy of the longitudinal record linkage. Our review included all client reports within each sampled eUCI40 and variables for matching and other support variables (e.g. the client enrollment status). To select our sample, we created stratification groups of eUCI40s that appeared in the RSR data for two, three, four, five, or six years. We then selected ten (10) eUCI40s from each stratification group, yielding a total sample of 50 eUCI40s. Each eUCI40 could be associated with multiple client records within and across years. If two or more of these records represented the same client, then the records were linked. This sample review only covered the 2010–2015 interval because only 2010–2010 RSR data were available when this review was conducted.

#### Comparison with an external data source

We also examined the extent to which our linked RSR records corresponded to records collected in RWHAP clinics. To accomplish this task, we used data from the HIV Research Network (HIVRN). HIVRN is a network of HIV care providers from across the United States who provide timely demographic, clinical, and health services costs and utilization data on people with HIV from a consortium of adult and pediatric clinics across the United States [50]. HIVRN data are close to an ideal data set for comparison because most of the HIVRN providers are funded by RWHAP and all clients in the network were uniquely identified across providers and years. We obtained aggregated provider-level data from the eleven service providers that are both in the HIVRN health care network and are funded through RWHAP. The final data for comparison contain information on the number of clients within the service provider each year, and the number of new clients within the service provider in both the current and prior year for calendar years 2010 through 2015. Since we only obtained the 2010 to 2015 aggregated provider-level data from the HIVRN, the validations only covered the 2010–2015 interval.

We used the HIVRN-RWHAP linkage ratio across all eleven service providers as a measure of comparison between these two data sets. The ratio is defined as:

$$\frac{\%\;of\;RWHAP\;clients\;found\;in\;both\;the\;current\;and\;previous\;analysis\;year\;}{\%\;of\;HIVRN\;clients\;found\;in\;both\;the\;current\;and\;previous\;analysis\;year\;}$$

A client was deemed “year-to-year linked” if they received care in both the previous year and the current year. The denominator of “% of clients found in both the current and previous analysis year” was the number of clients who received care in the previous analysis year. The rate of carryover from year to year, though not an explicit part of threshold selection or external validation methods, had a crucial role in both as a reality check. Linkage resulting in too low or too high a carryover rate would imply increases or decreases in numbers of clients that would be inconsistent with overall service levels and payments to providers.

## Results

### RSR linkage

Table 1 shows the results of the longitudinal linkage across the seven cycles from 2010–2016. Seventy percent of clients who appeared in 2010 also appeared in 2011. Seventy-four percent of clients who appeared in 2012 also appeared in either 2010 or 2011, or in both. In the year 2011, 30 percent of clients had not been seen in 2010, and in 2012, 26 percent of clients had not been seen in 2010 and 2011. In 2011, about 30 percent of clients had appeared in 2010 but were not found in 2011.

**Table 1:** RSR linkage results by year.

	# Clients Appearing in Current Year and Previous Years*	# of Clients Appearing in Current Year but not Previous Years**	# of Clients Appearing in Previous Year but Not Current Year***	# of Unique Clients Within Current Year
2010	-	-	-	
2011	385,753 (70%)	168,893 (30%)	170,422 (31%)	554,646
2012	395,152 (74%)	141,067 (26%)	176,263 (32%)	536,219
2013	394,801 (75%)	129,874 (25%)	168,726 (31%)	524,675
2014	407,267 (80%)	104,947 (20%)	146,650 (28%)	512,214
2015	423,625 (79%)	110,127 (21%)	128,138 (25%)	533,752
2016	437,456 (79%)	114,108 (21%)	127,271 (24%)	551,564

*The second column is the total number of clients who appeared in the current year and earlier years.

**The third column in this table is the total number of clients who appeared in the previous year but not in the current year.

***The fourth column shows the total of unique clients per year.

Table 2 provides information on the overall number of RWHAP clients who were linked using the longitudinal linkage. Overall, 39.5 percent of clients only appeared in one year; 49.6 percent of clients appeared in multiple years, but not all seven years; and 10.9 percent of clients appeared in all seven years.

**Table 2:** RSR overall linkage rate (2010–2016).

	# of Clients	Percent of Clients
**Clients only appearing in one year (2010–2016)**	**524,286**	**39.5**
**Clients appearing in multiple years**	**802,686**	**60.5**
Clients appearing in multiple years, but not all seven years	657,869	49.6
Clients appearing in all seven years	144,817	10.9

The total number of clients who appeared in multiple years was 802,686, representing about 60 percent of the total number of clients over the seven-year study.

### Validation

#### Sample review

The longitudinal linkage worked well among all sampled cases except for two cases in the 2014 cycle. Forty-eight of the fifty (96 percent) eUCI40 blocks had correct assignments of client records. Following annual deduplication in 2014, two cases previously matched on identifiers that included geographic unit codes failed to link after collection of the client location ended due to stricter privacy constraints.

#### Comparison to HIVRN data

Table 3 shows the 2010–2015 year-to-year HIVRN-RWHAP linkage ratio for the eleven matched service providers. The overall year-to-year client carry over rate for the HIVRN sites was about 84 percent, while the annual RSR longitudinal client carry over rate fluctuated and improved between 2010–2015 (from 67.7 to 78.6 percent). The range of the year-to-year HIVRN-RWHAP linkage ratio was from 79.9 to 94.2 percent. The RWHAP and RWHAP/HIVRN Ratio have slight upward trends after the first year of linkage.

**Table 3:** Year-to-year HIVRN-RWHAP linkage ratio from 11 matched service providers, 2010–2015.

Year	HIVRN % Linked*	RWHAP % Linked**	RWHAP-HIVRN Ratio
2010–2011	82.8	75.9	91.6
2011–2012	84.7	67.7	79.9
2012–2013	84.2	67.8	80.5
2013–2014	84.7	77.4	91.4
2014–2015	83.4	78.6	94.2

*HIVRN % Linked refers to the percent of HIV clients found in both analysis years, according to the provider’s records.

**RWHAP % Linked refers to the percent of HIV clients found in both analysis years, according to the F-S linkage methodology executed on anonymized data supplied to RWHAP by providers.

## Discussion

In the current paper, we describe the development of a longitudinal record linkage methodology for use with de-identified longitudinal data on PLWH. This methodology addresses a previous gap in data analytic capabilities by allowing HRSA HAB to link RWHAP clients across reporting years. Initial validation efforts suggest that this proposed methodology is strong in its ability to identify the same client across multiple reporting years. This longitudinal linkage methodology also will allow HRSA HAB to better evaluate patterns of viral suppression and service utilization over time. These types of analyses will support future activities to better understand the impact of the RWHAP on HIV/AIDS outcomes and will assist HRSA HAB in monitoring progress toward meeting National HIV/AIDS Strategy goals.

Although there were some noted differences in the comparison between HIVRN and RWHAP longitudinal linkage rates, these differences were not unexpected. The de-identified nature of the RSR data prevented a direct linkage of patients receiving care in the HIVRN network compared to RWHAP clients. Instead, we relied on cross sectional information about the RWHAP funded service providers in the HIVRN network. Some patients who received care at the HIVRN sites may not have been eligible to receive RWHAP services or did not receive RWHAP-funded services in the year. As such, these clients would not be reported in the RSR, but still would have been included in the HIVRN data set. Despite these differences, however, the improvements in the critical carryover rate from one year to another among RWHAP clients (Table 3) indicates improvements in longitudinal linkage during the 2010–2015 interval. Some of the difference between the HIVRN and RWHAP linkage rates do not appear to be due to RWHAP linkage failures alone. While over 50 percent of clients in the 2010–2016 longitudinal database appear consecutively in time spans of at least three years, the remainder appear in sequences with gaps that suggest changes in the RWHAP client or provider populations.

There are some important limitations to our approach that should be noted. Because RSR data do not include any client identification information (i.e., client first and last name, date of birth, and social security number), the quality of the linkage relies heavily on the quality of the eUCI40 and completeness of the client level data submitted to HRSA HAB annually by each provider. A client may receive multiple eUCI40s if the client’s identifying information was recorded differently across providers or across years. If so, this client’s records would never be matched using our algorithm. This is also true for our within-year linkages. Additionally, our linkage methodology is more likely to miss clients who moved across states and CBSA and were served by an entirely new list of providers. Unfortunately, the extent to which this linkage methodology may miss clients who have moved to other geographic areas is unknown. If attributes of these individuals are associated with lower rates of viral suppression or access to care, subsequent results from analyses of these outcomes using RSR data could be biased. Nevertheless, the proportion of clients who fall into this category could be small. While many factors associated with HIV risk (e.g., homelessness) are linked to high mobility, studies suggest that residential mobility, particularly among the poor, are often short distances as opposed to moving out of state [51–54].

The development of this longitudinal linkage methodology has important implications in the fields of public health and health services research [51]. The linkage of multiple administrative and/or electronic health records can provide valuable opportunities to answer research questions that are not possible with single data sources alone. Nevertheless, there are often data sharing restrictions between and across the organizations that collect these data, particularly when data are contained in medical records. Even if there are no such restrictions on sharing data, laws and regulations, such as the Social Security Number Fraud Prevention Act of 2017, Privacy Act of 1974, HIPPA, Health Insurance Portability and Accountability Act, prohibit the sharing of personal identifiers that can be used to link the data. As a result, there is an important need for alternative methods to facilitate accurate linkages of health records in the absence of personal identifiers.

## Supporting information

S1 Appendix (DOCX)
