# Supplementary material for: Methodology for linking Ryan White HIV/AIDS Program Services Report (RSR) client level data over multiple years
Source: PLoS One. 2020 Aug 21;15(8):e0237635. doi: 10.1371/journal.pone.0237635 (PMC7442495; doi:10.1371/journal.pone.0237635)
Supplement: S1 Appendix — (DOCX) [file pone.0237635.s001.docx]

**APPENDIX I: BIBLIOGRAPHY-** **RECORD LINKAGE ALGORITHMS BY TYPE**

**Foundations of data/record linkage**

1. Newcombe HB, Kennedy JM, Axford SJ, James AP. Automatic linkage of vital records. Science (New York, NY). 1959;130(3381):954-9.
2. Newcombe HB, Kennedy JM. Record linkage: making maximum use of the discriminating power of identifying information. Commun ACM. 1962;5(11):563–6.
3. Fellegi IP, Sunter AB. A Theory for Record Linkage. Journal of the American Statistical Association. 1969;64(328):1183-210.
4. Copas JB, Hilton FJ. Record Linkage: Statistical Models for Matching Computer Records. Journal of the Royal Statistical Society Series A (Statistics in Society). 1990;153(3):287-320.
5. Jaro MA. Probabilistic linkage of large public health data files. Statistics in medicine. 1995;14(5-7):491-8.
6. Belin TR, Rubin DB. A Method for Calibrating False-Match Rates in Record Linkage. Journal of the American Statistical Association. 1995;90(430):694-707.
7. Brenner H, Schmidtmann I, Stegmaier C. Effects of record linkage errors on registry-based follow-up studies. Statistics in medicine. 1997;16(23):2633-43.
8. Porter EH, Winkler WE, editors. Approximate string comparison and its effect on an advanced record linkage system. Advanced record linkage system US Bureau of the Census, Research Report; 1997: Citeseer.
9. McCallum A, Nigam K, Ungar LH. Efficient clustering of high-dimensional data sets with application to reference matching. Proceedings of the sixth ACM SIGKDD international conference on Knowledge discovery and data mining; Boston, Massachusetts, USA: Association for Computing Machinery; 2000. p. 169–78.
10. Winkler W, Gov W. Using the EM Algorithm for Weight Computation in the Fellegi-Sunter Model of Record Linkage. Journal of the American Statistical Association. 2002.
11. Gill L. Methods for automatic record matching and linkage and their use in national statistics. Office for National Statistics; 2001.
12. Fortini M, Liseo B, Nuccitelli A, Scanu M. On Bayesian record linkage. Research in Official Statistics. 2001;4(1):185-98.
13. Gomatam S, Carter R, Ariet M, Mitchell G. An empirical comparison of record linkage procedures. Statistics in medicine. 2002;21(10):1485-96.
14. Churches T, Christen P. Blind data linkage using n-gram similarity comparisons. In Pacific-Asia Conference on Knowledge Discovery and Data Mining. Springer, Berlin, Heidelberg. 2004:121-6.
15. Larsen MD. Advances in record linkage theory: Hierarchical Bayesian record linkage theory. In Proceedings of the Section on Survey Research Methods. 2005:3277-84.
16. Herzog TN, Scheuren FJ, Winkler WE. Data quality and record linkage techniques: Springer Science & Business Media; 2007.
17. DuVall SL, Kerber RA, Thomas A. Extending the Fellegi-Sunter probabilistic record linkage method for approximate field comparators. Journal of Biomedical Informatics. 2010;43(1):24-30.
18. Randall SM, Ferrante AM, Boyd JH, Semmens JB. The effect of data cleaning on record linkage quality. BMC Medical Informatics and Decision Making. 2013;13(1):64.
19. Harron K, Goldstein H, Dibben C. Methodological developments in data linkage. Chichester, West Sussex, United Kingdom: John Wiley & Sons Inc.; 2016.

**Efficient large-scale data/record linkage methods and techniques**

1. Baxter R, Christen P, Churches T, editors. A comparison of fast blocking methods for record linkage. Proc ACM SIGKDD'03 Workshop on Data Cleaning, Record Linkage, and Object Consolidation; 2003; Washington, DC.
2. Yancey WE. BigMatch: A program for extracting probable matches from a large file for record linkage. Computing. 2002;1(1):1-8.
3. Gu L, Baxter R. Adaptive filtering for efficient record linkage. In Proceedings of the 2004 Society for Industrial and Applied Mathematics International Conference on Data Mining; 2004: SIAM..
4. Bilenko M, Kamath B, Mooney RJ, editors. Adaptive blocking: Learning to scale up record linkage. Sixth International Conference on Data Mining (ICDM'06); 2006: IEEE.
5. Christen P. Towards Parameter-free Blocking for Scalable Record Linkage. Canberra, ACT: Dept. of Computer Science, Faculty of Engineering and Information Technology, The Australian National University; 2007.
6. Hermansen SW, Leitzmann MF, Schatzkin A. The impact on National Death Index ascertainment of limiting submissions to Social Security Administration Death Master File matches in epidemiologic studies of mortality. American journal of epidemiology. 2009;169(7):901-8.
7. Winkler WE, Yancey WE, Porter EH. Fast Record Linkage of Very Large Files in Support of Decennial and Administrative Records Projects. Proceedings of the Section on Survey Research Methods, American Statistical Association, Alexandria, VA. 2010.
8. Christen P. A survey of indexing techniques for scalable record linkage and deduplication. IEEE transactions on knowledge and data engineering. 2011 Jun 16;24(9):1537-55.
9. McNeill N, Kardes H, Borthwick A. Dynamic record blocking: efficient linking of massive databases in MapReduce. In Proceedings of the 10th International Workshop on Quality in Databases (QDB) 2012.
10. Wagner D, Lane M. The Person Identification Validation System (PVS): Applying the Center for Administrative Records Research and Applications’ (CARRA) Record Linkage Software. Center for Economic Studies, Washington, DC: US Census Bureau. 2014. Available from <https://ideas.repec.org/p/cen/cpaper/2014-01.html>.
11. Steorts RC, Ventura SL, Sadinle M, Fienberg SE. A comparison of blocking methods for record linkage. In International Conference on Privacy in Statistical Databases (PSD); Springer, Cham. 2014:253-68.
12. Aleshin-Guendel S, Sadinle M. Multiﬁle Record Linkage and Duplicate Detection Via a Structured Prior for Partitions July 30, 2019, slides available from lead author.

**Secure, privacy-preserving data/record linkage**

1. Churches T, Christen P. Some methods for blindfolded record linkage. BMC Medical Informatics and Decision Making. 2004;4(1):9.
2. Elamir EA, Skinner C. Record Level Measures of Disclosure Risk for Survey Microdata. Journal of Official Statistics. 2006;22(3):525-39.
3. Schnell R. Privacy-preserving record linkage and privacy-preserving blocking for large files with cryptographic keys using multibit trees. JSM Proceedings, Survey Research Methods Section. 2013:187-94.
4. Schnell R, Bachteler T, Reiher J. Privacy-preserving record linkage using Bloom filters. BMC Medical Informatics and Decision Making. 2009;9(1):41.
5. Verykios VS, Karakasidis A, Mitrogiannis VK. Privacy preserving record linkage approaches. International Journal of Data Mining, Modelling and Management. 2009;1(2):206-21.

**Statistical/machine learning and AI linkage methods**

1. Winkler WE. Machine learning, information retrieval and record linkage. In Proc Section on Survey Research Methods, American Statistical Association. 2000:20-9.
2. Bilenko M, Mooney R, Cohen W, Ravikumar P, Fienberg S. Adaptive name matching in information integration. IEEE Intelligent Systems. 2003;18(5):16-23.
3. Domingo-Ferrer J, Torra V. On the connections between statistical disclosure control for microdata and some artificial intelligence tools. Information Sciences. 2003;151:153-70.
4. Michelson M, Knoblock CA. Learning blocking schemes for record linkage. In AAAI 2006;6: 440-5.
5. Wilson DR. Beyond probabilistic record linkage: Using neural networks and complex features to improve genealogical record linkage. In The 2011 International Joint Conference on Neural Networks. IEEE. 2011:9-14.
6. Giang PH. A machine learning approach to create blocking criteria for record linkage. Health Care Management Science. 2015;18(1):93-105.
7. Wang F, Wang H. Record linkage using the combination of twice iterative svm training and controllable manual review. In 2016 IEEE 14th Intl Conf on Dependable, Autonomic and Secure Computing, 14th Intl Conf on Pervasive Intelligence and Computing, 2nd Intl Conf on Big Data Intelligence and Computing and Cyber Science and Technology Congress (DASC/PiCom/DataCom/CyberSciTech) IEEE. 2016:31-8.
8. Enamorado T. Active Learning for Probabilistic Record Linkage. Available at SSRN 3257638. 2018.
9. Hejblum BP, Weber GM, Liao KP, Palmer NP, Churchill S, Shadick NA, Szolovits P, Murphy SN, Kohane IS, Cai T. Probabilistic record linkage of de-identified research datasets with discrepancies using diagnosis codes. Scientific Data. 2019;6:180298.
10. Comber S, Arribas‐Bel D. Machine learning innovations in address matching: A practical comparison of word2vec and CRFs. Transactions in GIS. 2019;23(2):334-48.
11. Acheson E, Volpi M, Purves RS. Machine learning for cross-gazetteer matching of natural features. International Journal of Geographical Information Science. 2020;34(4):708-34.

**Data/record linkage and disclosure risk**

1. Lambert D. Measures of disclosure risk and harm. Journal of Official Statistics. 1993;9:313-31.
2. Winkler WE. Producing public-use microdata that are analytically valid and confidential. Washington, DC: US Bureau of the Census; 1998.
3. Sweeney L. Computational disclosure control for medical microdata: The Datafly system. In Record Linkage Techniques 1997: Proceedings of an International Workshop and Exposition 1997:442-53).
4. Winkler WE. Re-identification methods for evaluating the confidentiality of analytically valid microdata. Statistics. 2005;9:1-4.
5. Quantin C, Bouzelat H, Allaert FA, Benhamiche AM, Faivre J, Dusserre L. How to ensure data security of an epidemiological follow-up: quality assessment of an anonymous record linkage procedure. International journal of medical informatics. 1998;49(1):117-22.
6. Abowd JM, Woodcock SD. Disclosure limitation in longitudinal linked data. Confidentiality, Disclosure, and Data Access: Theory and Practical Applications for Statistical Agencies. 2001;215277.
7. Malin B, Sweeney L, Newton E. Trail re-identification: learning who you are from where you have been. Proc. LIDAP-WP12. 2003.
8. Agrawal R, Evfimievski A, Kiernan J, Velu R. Auditing disclosure by relevance ranking. In Proceedings of the 2007 ACM SIGMOD International Conference on Management of Data 2007:79-90.
9. Alleva G, Fortini M, Tancredi A. The control of non-sampling errors on linked data: An application on population census. In Proceedings of the 2007 Intermediate Conference. Risk and Prediction. Venice. 2007.
10. Skinner C, Shlomo N. Assessing identification risk in survey microdata using log-linear models. Journal of the American Statistical Association. 2008;103(483):989-1001.
11. Shlomo N, Skinner C. Assessing the disclosure protection provided by misclassification for survey microdata. Working Paper M09/14. Southampton Statistical Sciences Research Institute. 2009.
12. Hall R, Fienberg SE. Privacy-preserving record linkage. In Domingo-Ferrer J, Magkos E, editors. Privacy in Statistical Databases: UNESCO Chair in Data Privacy, International Conference, PSD 2010, Corfu, Greece, September 22-24, 2010, Proceedings. Springer Science & Business Media; 2010 Sep 9.
13. Kuzu M, Kantarcioglu M, Durham E, Malin B. A constraint satisfaction cryptanalysis of Bloom filters in private record linkage. In International Symposium on Privacy Enhancing Technologies Symposium. Springer, Berlin, Heidelberg. 2011:226-245.
14. Schnell R, Bachteler T, Reiher J. A novel error-tolerant anonymous linking code. 2011. Available at SSRN 3549247.
15. Vatsalan D, Christen P, Verykios VS. A taxonomy of privacy-preserving record linkage techniques. Information Systems. 2013;38(6):946-69.

**Data quality, analysis of linked data and bias**

1. Neter J, Maynes ES, Ramanathan R. The effect of mismatching on the measurement of response errors. Journal of the American Statistical Association. 1965;60(312):1005-27.
2. Howe GR, Lindsay J. A generalized iterative record linkage computer system for use in medical follow-up studies. Computers and Biomedical Research. 1981;14(4):327-40.
3. Armstrong JB, Mayda JE. Model-based estimation of record linkage error rates. Survey Methodology. 1993;19(2):137-47.
4. Bartlett S, Krewski D, Wang Y, Zielinski JM. Evaluation of error rates in large scale computerized record linkage studies. Survey Methodology. 1993;19(1):3-12.
5. Scheuren F, Winkler WE. Regression analysis of data files that are computer matched – Part I. Survey Methodology, 1993;19:39-58.
6. Winkler W, Scheuren F. Recursive analysis of linked data files. In Proceedings of the 1996 Census Bureau Annual Research Conference 1996.
7. Scheuren F, Winkler WE. Recursive merging and analysis of administrative lists and data. Proceedings of the Section of Government Statistics, American Statistical Association, US Bureau of the Census; 1996:123–8 .
8. Scheuren F, Winkler WE. Regression analysis of data files that are computer matched – Part II. Survey Methodology. 1997;23:157-65.
9. Hernández MA, Stolfo SJ. Real-world data is dirty: Data cleansing and the merge/purge problem. Data mining and knowledge discovery. 1998;2(1):9-37.
10. Bigelow W, Karlson T, Beutel P. Using probabilistic linkage to merge multiple data sources for monitoring population health. In Association for Health Services Research Meetings. Madison, WI. 1999;16:4-5.
11. Lahiri P, Larsen MD. Model-based analysis of records linked using mixture models. In Proceedings of the American Statistical Association, Survey Research Methods Section. 2000:11-9.
12. Liseo B, Tancredi A. Statistical inference for data files that are computer linked. In Proceedings of the International Workshop on Statistical Modelling, Firenze Univ. Press. 2004:224-8.
13. Lahiri P, Larsen MD. Regression analysis with linked data. Journal of the American Statistical Association. 2005;100(469):222-30.
14. Elmagarmid AK, Ipeirotis PG, Verykios VS. Duplicate record detection: A survey. IEEE Transactions on Knowledge and Data Engineering. 2007;19(1):1-16.
15. Fienberg SE, Manrique-Vallier D. Integrated methodology for multiple systems estimation and record linkage using a missing data formulation. Advances in Statistical Analysis. 2009;93(1):49-60.
16. Winkler, WE. Very fast methods of cleanup and statistical analysis of national files. Proceedings of the Section on Survey Research Methods, American Statistical Association, CD-ROM. 2014.
17. National Center for Health Statistics. The linkage of National Center for Health Statistics survey data to the National Death Index—2015 linked mortality file (LMF): methodology overview and analytic considerations. Retrieved May 2019. https://www.cdc.gov/nchs/data-linkage/mortality-methods.htm.
